# Location-specific technical nuances of spinal meningioma resection: an operative video case series

**DOI:** 10.3171/2023.7.FOCVID2339

**Published:** 2023-10-01

**Authors:** Wilson A. M. Fisher, Cheerag Upadhyaya, Michael Galgano

**Affiliations:** 1 University of North Carolina School of Medicine, Chapel Hill; and; 2 Department of Neurosurgery, University of North Carolina, Chapel Hill, North Carolina

**Keywords:** intradural tumor, cervical myelopathy, spine, meningioma

## Abstract

The objective of this video was to demonstrate technical nuances of intradural spinal meningioma (ISM) resection through a high-quality surgical video. The authors describe 3 patients with ISM in the cervicomedullary, cervical, and thoracic regions. Patients underwent surgery in the prone position with laminectomy, dorsal durotomy, and then resection of the mass. Case 1 required a suboccipital craniectomy and dissection of the tumor away from the vertebral artery. In case 2, special emphasis is placed on sectioning the dentate ligament with cord rotation. Case 3 highlights meticulous circumferential arachnoid release and the use of ultrasound. Patients saw significant neurological improvement postoperatively. This video provides clear instruction on location-specific technical nuances of ISM removal.

## Transcript

### 0:21 Case 1: Clinical Information, Imaging, and Surgical Plan.

This patient is a 60-year-old female presenting with cervical myelopathy. Preoperative imaging reveals a large, calcified mass at the cranial-vertebral junction.

### 0:32 Preoperative CTA.

A preoperative CTA reveals the relationship of the meningioma to the posterior circulation.

### 0:39 Surgical Plan.

Our surgical plan will be detailed in the video to follow.

### 0:44 Craniectomy and Laminectomy.

After standard posterior cervical exposure, we performed a suboccipital craniectomy and C1 laminectomy.^1–5^ Ultrasonography revealed the intradural nature of the mass.

### 0:57 Dural Incision.

The dura is then opened with an 11 blade scalpel. Dural tack-up sutures are placed and tethered to the posterior paraspinal musculature.

### 1:06 Tumor Mobilization and Excision.

After superficial exposure of the tumor, color flow Doppler aided in the identification of the vertebral artery. We begin by using bipolar cautery to dissect the tumor away from the right side of the dura. This is a critical step as it is also used to devascularize the tumor. A Mott dissector is then used to further peel the tumor away from the dura. Although not shown in this video, direct stimulation was used to identify the 11th cranial nerve. At this stage, we carefully begin to mobilize the upper pole of the tumor. Further dissection allows us to remove the detached component of the tumor.

### 1:50 Retensioning of Dural Tack-Up Sutures.

Throughout the duration of the operation, it is important to frequently retension the dural tack-up sutures. This maneuver allows for continued traction and countertraction of the mass. We aim to expose the caudal end of the tumor.

### 2:04 Arachnoid Dissection.

We use microscissors to dissect and section the distal arachnoid as well as the dentate ligaments. The arachnoid dissection is carried in the rostral direction. We then further mobilize the tumor.

### 2:20 Tumor Delivery.

The majority of the tumor can then be subsequently delivered with a Mott dissector.

### 2:25 Residual Tumor Removal.

Once the bulk of the tumor is delivered, we can turn our attention to the remaining mass that is tethered to the lateral dural margin as well as the vertebral artery. A small rind of tumor was left on the medial border of the vertebral artery to avoid iatrogenic injury. The final component of the tumor is then removed after sectioning the remaining tethering points.

### 2:54 Neurovascular Anatomy Revealed.

We can now clearly appreciate PICA (posterior inferior cerebellar artery), the vertebral artery, spinal accessory nerve, and filaments of the C2 nerve root.

### 3:04 Closure.

The dura is then closed with a running 6-0 Prolene suture. Fibrin glue is then injected over the suture line for extra reinforcement.

### 3:15 Postoperative Summary.

The patient’s immediate postoperative course was complicated by a symptomatic pseudomeningocele. This ultimately required a revision dural closure and temporary spinal fluid diversion. During the revision operation, a small spinal fluid leak was appreciated at the top end of the dural closure. A muscle pledget was sutured over the defect, followed by application of fibrin glue. Postoperative MRI scan revealed a gross-total resection of the meningioma. The patient continues to have resolution of her preoperative myelopathic symptoms 5 months after surgery.

### 3:50 Case 2: Clinical Information, Imaging, and Surgical Plan.

The second patient in our case series is a 71-year-old female presenting with left-sided hemiparesis and the large ventral cervical mass centered in the subaxial spine. The surgical plan will be detailed in the video to follow.

### 4:06 Patient Positioning.

It should be noted that preoperative upright x-rays revealed a high T1 slope. We felt that a fusion was necessary to allow the patient the ability to continue achieving a commensurate amount of cervical lordosis after a three-level laminectomy. It should be noted that prone positioning initiated a significant drop in motor evoked potentials on the left side. A slight flexion maneuver of the head yielded return of our lost electrophysiological signals.

### 4:36 Surgical Video Start.

After standard posterior neck exposure, we place instrumentation from C2 to C6. Left-sided medial facetectomies are then completed. A 4-mm round, coarse diamond drill bit is then further used to make bilateral troughs. Posterior elements are then removed and used for autograft later in the case.

### 4:59 Dorsal Durotomy.

An 11 blade is then used to make a dorsal lateral durotomy on the left side. A dental tool is placed underneath the dura during the dural opening to protect the nerve roots and underlying spinal cord. We then perform our rostral and caudal arachnoid dissection using microscissors.

### 5:19 Sectioning of the Dentate Ligament.

The dentate ligament is then subsequently sectioned. A suture can be placed through the dentate ligament. This allows mobilization and gentle rotation of the spinal cord to give the surgeon access to the ventral intradural compartment. For select ventral intradural tumors that span multiple segments, more than one dentate ligament may need to be sectioned. Neural monitoring signals should frequently be checked during rotation of the spinal cord.

### 5:53 Tumor Mobilization.

A half-by-half cottonoid is then placed at the rostral end of the tumor. Tumor dissectors are used to further mobilize the meningioma away from the dura. A second cottonoid is placed in the caudal end of the tumor. We now subsequently begin the process of tumor mobilization. We use a combination of blunt and sharp dissection. Frequent bipolar cautery is used on the surface of the tumor as well as the dural margins to devascularize and shrink the tumor. This process is continued until the meningioma reaches a more manageable size. We use dynamic retraction to move the meningioma away from the spinal cord. The bipolar cautery is then used to further devascularize and dissect the tumor away from the ventral dural margin. This particular tumor dissection technique is in contrast to piecemeal resection of the mass. After the superior and ventral margins of the tumor are completely dissected, we now peel away the caudal end of the tumor from the dura.

### 7:10 Tumor Removal.

After all sides of the meningioma have been adequately dissected and released, the mass can be removed en bloc. A Woodson elevator is then placed in the ventral intradural compartment to ensure that there is no further palpable tumor on the contralateral side.

### 7:29 Closure and Patient Repositioning.

The dura is then closed with a 4-0 running Nurolon. The head is then repositioned into neutral alignment prior to rod insertion.

### 7:37 Postoperative Summary.

We were able to achieve a gross-total resection of the meningioma. At 18-month follow-up, the patient no longer has left-sided hemiparesis and is able to ambulate unassisted.

### 7:47 Case 3: Clinical Information, Imaging, and Surgical Plan.

The last patient in our case series is a 69-year-old female presenting with 3 months of progressive lower-extremity weakness with bowel and bladder incontinence. She was found to have an enhancing mass in the intradural compartment at the level of T6/T7 consistent with a meningioma. The surgical plan will be detailed in the video to follow.

### 8:10 Intraoperative Ultrasound.

After a laminectomy is performed at T6/T7, an ultrasound can be brought into the operative field. The mass can clearly be identified in addition to arachnoid loculations.

### 8:21 Dural Incision.

The dura is then opened in standard fashion.

### 8:25 Arachnoid Dissection.

After dural tack-up sutures are placed, we begin our arachnoid dissection using a combination of fine jeweler forceps and microscissors. To avoid unwanted iatrogenic injury to the spinal cord, it is critically important to perform a circumferential arachnoid release. Neglecting to perform this critical maneuver can result in undue traction on the spinal cord with any manipulation of the tumor. It can be appreciated that dorsal thoracic nerve rootlets are draped over the tumor. Such nerve rootlets can safely be cauterized and divided. Microscissors are then used to perform further ventral arachnoid dissection. This aids in mobilization of the tumor.

### 9:17 Dissection of Meningioma From Lateral Margin of Spinal Cord.

The most challenging component of this operation is dissecting the meningioma away from the lateral margin of the spiral cord. A combination of blunt and sharp dissection is used to further mobilize the tumor away from the spinal cord.

### 9:34 Emphasis of Full Dissection Prior to Tumor Removal.

At this point of the operation, it is critically important that the surgeon resists the urge to pull the tumor away from the spinal cord. Doing so can result in a loss of neural monitoring signals with subsequent spinal cord injury. The surgeon must remain patient during this part of the operation and continue meticulous dissection of the arachnoid off the tumor. Doing so will allow the tumor to easily be mobilized away from the spinal cord. We intermittently utilize bipolar cautery on the tumor. This further emulsifies and shrinks the tumor. Doing so makes it easier to peel the meningioma away from the spinal cord.

### 10:17 Final Dissection of Meningioma.

The last remaining attachments of the tumor are dissected away. The final tethering points of the tumor are then cauterized and divided.

### 10:29 En Bloc Tumor Resection.

En bloc delivery of the tumor can then be achieved.

### 10:35 Contralateral Arachnoid Release.

Now that the tumor has completely been resected, we can perform a contralateral arachnoid release. This allows us to transpose the spinal cord back to a more anatomical position.

### 10:53 Closure.

The dura is then closed in usual fashion. Intraoperative ultrasound further confirmed a gross-total resection of the meningioma. Fibrin glue is then injected over the suture line.

### 11:04 Postoperative Summary.

A gross-total resection was achieved. At 5-month follow-up, the patient is now full strength in the bilateral lower extremities. She is now able to ambulate unassisted and is experiencing resolution of her bowel and bladder dysfunction.

## Disclosures

The authors report no conflict of interest concerning the materials or methods used in this study or the findings specified in this publication.

## Author Contributions

Primary surgeon: Galgano. Editing and drafting the video and abstract: Fisher, Galgano. Critically revising the work: Fisher, Galgano. Reviewed submitted version of the work: all authors. Approved the final version of the work on behalf of all authors: Fisher. Supervision: Fisher, Galgano.

## Supplemental Information

### Patient Informed Consent

The necessary patient informed consent was obtained in this study.
